# Melatonin Mitigates Drought Induced Oxidative Stress in Potato Plants through Modulation of Osmolytes, Sugar Metabolism, ABA Homeostasis and Antioxidant Enzymes

**DOI:** 10.3390/plants11091151

**Published:** 2022-04-24

**Authors:** Ahmed Abou El-Yazied, Mohamed F. M. Ibrahim, Mervat A. R. Ibrahim, Ibrahim N. Nasef, Salem Mesfir Al-Qahtani, Nadi Awad Al-Harbi, Fahad Mohammed Alzuaibr, Abdullah Alaklabi, Eldessoky S. Dessoky, Nadiyah M. Alabdallah, Mohamed M. A. Omar, Mariam T. S. Ibrahim, Amr A. Metwally, Karim. M. Hassan, Said A. Shehata

**Affiliations:** 1 Department of Horticulture, Faculty of Agriculture, Ain Shams University, Cairo 11566, Egypt; ahmed_abdelhafez2@agr.asu.edu.eg (A.A.E.-Y.); amr_metwally@agr.asu.edu.eg (A.A.M.); kareem_hassan@agr.asu.edu.eg (K.M.H.); 2 Department of Agricultural Botany, Faculty of Agriculture, Ain Shams University, Cairo 11566, Egypt; said_shehata@agr.asu.edu.eg; 3 Department of Biochemistry, Faculty of Agriculture, Ain Shams University, Cairo 11566, Egypt; mervat_ibrahim@agr.asu.edu.eg (M.A.R.I.); mohamed.omar@agr.asu.edu.eg (M.M.A.O.); mariam_ibrahim@agr.asu.edu.eg (M.T.S.I.); 4 Department of Horticulture, Faculty of Agriculture, Suez Canal University, Ismailia 41522, Egypt; innasef@agr.suez.edu.eg; 5 Biology Department, University College of Tayma, Tabuk University, P.O. Box 741, Tabuk 47512, Saudi Arabia; salghtani@ut.edu.sa (S.M.A.-Q.); nalharbi@ut.edu.sa (N.A.A.-H.); 6 Department of Biology, Faculty of Science, University of Tabuk, P.O. Box 741, Tabuk 71491, Saudi Arabia; falzuaiber@ut.edu.sa; 7 Department of Biology, Faculty of Science, University of Bisha, P.O. Box 551, Bisha 61922, Saudi Arabia; alaklabia@gmail.com; 8 Department of Biology, College of Science, Taif University, P.O. Box 11099, Taif 21944, Saudi Arabia; es.dessouky@tu.edu.sa; 9 Department of Biology, College of Science, Imam Abdulrahman Bin Faisal University, P.O. Box 1982, Dammam 31441, Saudi Arabia; nmalabdallah@iau.edu.sa

**Keywords:** *solanum tuberosum* L., carbonyl stress, alpha-ketoaldehyde methylglyoxal, tuberization and water stress

## Abstract

The effect of melatonin (MT) on potato plants under drought stress is still unclear in the available literature. Here, we studied the effect of MT as a foliar application at 0, 0.05, 0.1, and 0.2 mM on potato plants grown under well-watered and drought stressed conditions during the most critical period of early tuberization stage. The results indicated that under drought stress conditions, exogenous MT significantly (*p* ≤ 0.05) improved shoot fresh weight, shoot dry weight, chlorophyll (Chl; a, b and a + b), leaf relative water content (RWC), free amino acids (FAA), non-reducing sugars, total soluble sugars, cell membrane stability index, superoxide dismutase (SOD), catalase (CAT), guaiacol peroxidase (G-POX), and ascorbate peroxidase (APX) compared to the untreated plants. Meanwhile, carotenoids, proline, methylglyoxal (MG), H_2_O_2_, lipid peroxidation (malondialdehyde; MDA) and abscisic acid (ABA) were significantly decreased compared to the untreated plants. These responses may reveal the protective role of MT against drought induced carbonyl/oxidative stress and enhancing the antioxidative defense systems. Furthermore, tuber yield was differentially responded to MT treatments under well-watered and drought stressed conditions. Since, applied-MT led to an obvious decrease in tuber yield under well-watered conditions. In contrast, under drought conditions, tuber yield was substantially increased by MT-treatments up to 0.1 mM. These results may imply that under water deficiency, MT can regulate the tuberization process in potato plants by hindering ABA transport from the root to shoot system, on the one hand, and by increasing the non-reducing sugars on the other hand.

## 1. Introduction

Drought stress or water scarcity is one of the most restricted factors to growth and productivity of several plant species [1,2,3]. It poses a serious threat to food security in many regions worldwide [4]. Moreover, frequent climatic change and global warming can cause severe increments in the rate of evapotranspiration [5]. In this context, it is expected that by 2050, drought stress will cause serious problems for more than 50% of arable lands [6]. In higher plants, drought stress can be involved in a wide spectrum of complex events at morphological, physiological, biochemical and molecular levels [7,8,9,10]. These influences include hindering of stomatal conductance, CO_2_ fixation, transpiration rate, nutrients’ transport, and restriction of electron transport chain (ETC) leading to a significant disturbance in photosynthesis and respiration [11,12,13,14]. Besides, drought stress affects hormonal balance, various signaling processes and collapses cell membranes’ structure and dysfunction [15,16,17,18]. All of these destructive effects are widely correlated with elevating the concentration of reactive oxygen species (ROS) which cause oxidative damages to protein, lipids, and nucleic acids [19].

Melatonin (MT; N-acetyl-5-methoxy-tryptamine) is a naturally synthesized compound in animals, plants and microbes [20]. It is able to delay senescence, stimulating root growth, regulation fruit ripening and protecting photosynthetic systems [21,22]. Moreover, it has been found that exogenous melatonin can improve plant tolerance to various abiotic stresses i.e., heat stress [23], chilling [24], heavy metals [25], salinity [26], and drought [27,28,29]. Since applied MT can prevent the oxido-nitrositive induced damages by reducing the cytotoxic effects of reactive oxygen and/or nitrogen species (ROS/RNS) [20]. Additionally, MT plays an important role in the methylglyoxal (MG) detoxification [30] and enhancing plant water relations by stimulating the osmolytes’ biosynthesis [27]. Furthermore, MT can reinforce the photosynthesis efficiency [31] by affecting the transcription key genes that are involved in chlorophyll metabolism [23], upregulating the enzymes of Calvin’s cycle [32] and maintaining the plant cell redox status [33]. Melatonin is also an important regulator of gene expression related to phytohormones and their metabolism in plants i.e., indole-3-acetic acid (IAA), GAs, cytokinins (CKs), ABA, jasmonic acid (JA), nitric oxide (NO), ethylene (C_2_H_4_) and salicylic acid [21].

Methylglyoxal is a reactive carbonyl species causing oxidative stress under various abiotic stresses [34]. It can also serve as a signaling molecule under stress responses and tolerance [35,36]. Under severe stress conditions, it is considered a toxic molecule that can restrict plant growth, developmental, photosynthesis, and seed germination, [36]. Methylglyoxal detoxification depends on two glyoxalase enzymes (Gly I and Gly II) which can be boosted by exogenous melatonin under abiotic stress conditions [30,37].

The potato (*Solanum tuberosum* L.) is one of the most valuable edible crops worldwide [38,39]. It is sensitive to drought stress during different growth stages and tuber development due to the shallow root system that reduces its ability for water uptake and speed recovery after stress conditions [3,40]. Tuber initiation (tuberization) is most critical growth stage in potato plants [41,42]. Tuberization is a complex developmental and physiological process in potato plants. It requires an increase in ABA and non-reducing sugars leading to inhibition of stolon elongation and starting of tuber formation [43]. Several previous studies revealed that tuberization in potato plants and productivity can be affected by exogenous compounds i.e., plant growth regulators, antioxidants and osmo-protectants under various abiotic stress conditions [44,45,46].

Generally, the effect of exogenous MT on potato tuberization under drought stress is still unclear in literatures. Therefore, we conducted this study to investigate the possible protective effects of exogenous MT on drought-stressed potato plants and to understand its relation to the tuberization process by regulating the endogenous ABA (the key plant hormone under drought stress and tuberization process) and maintaining the concentration of non-reducing sugars (the major form for sugar transport and starch synthesis in tubers).

## 2. Results

### 2.1. Effect of Melatonin on Plant Growth and Photosynthetic Pigments

**Figure 1:**
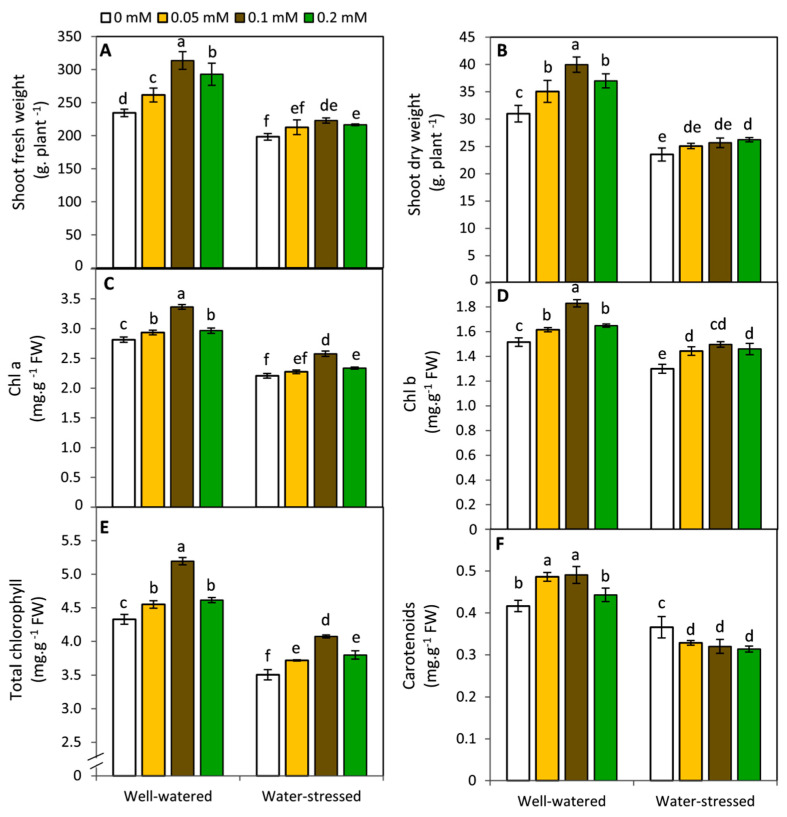
Effect of exogenous melatonin (MT; 0, 0.05, 0.1 and 0.2 mM) on shoot fresh weight (**A**), shoot dry weight (**B**), Chl a (**C**), Chl b (**D**), Chl a + b (**E**) and carotenoids (**F**) of potato plants grown under drought stress during the early stage of tuberization. Bars represent standard deviation (SD) of the means (*n* = 3). Different letters indicate significant differences among the treatments at *p* ≤ 0.05, according to Duncan’s multiple range test. Chl, chlorophyll.

Plants exposed to drought stress revealed a significant (*p* ≤ 0.05) decrease in growth parameters and photosynthetic pigments compared to the well-watered plants (Figure 1). However, exogenous MT enhanced shoot fresh weight, shoot dry weight, Chl a, Chl b and total chlorophyll in both water stressed and non-stressed plants. Generally, the highest significant results were obtained by the treatment of 0.1 mM MT in this respect. Conversely, MT treatments revealed divergent effects on carotenoids under water stressed and non-stressed conditions. In this context, carotenoids were significantly enhanced by MT treatments specifically at 0.05 and 0.1 mM under well-watered conditions. In contrast, under drought stress, all MT-treatments significantly reduced carotenoids compared to the untreated plants. These responses may imply that MT has a dual impact on the metabolism of terpenoids’ pathway (the major pathway of carotenoids and ABA biosynthesis in higher plants) in potato plants under sufficient and deficit water supply.

### 2.2. Effect of Melatonin on RWC and Osmolytes

**Figure 2:**
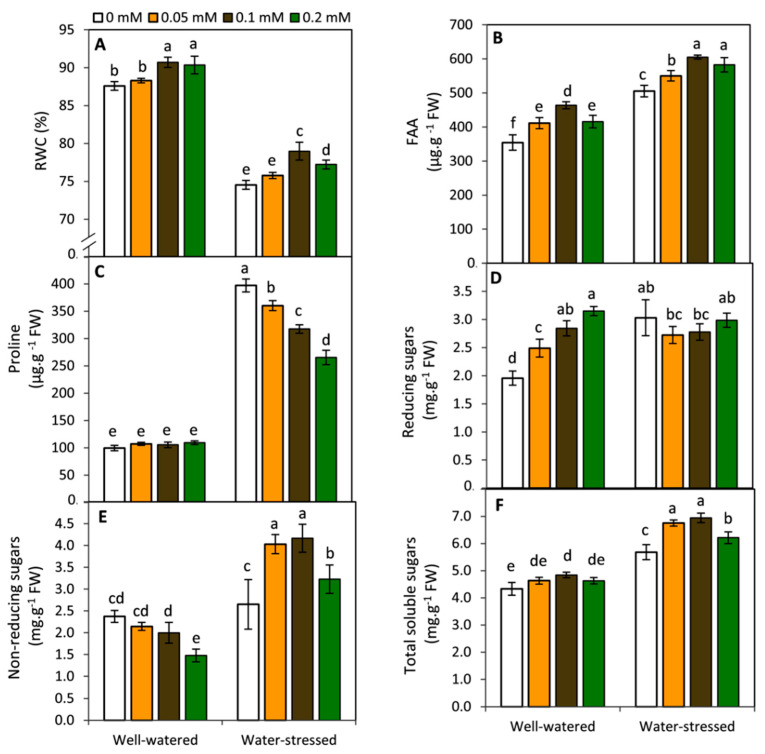
Effect of exogenous melatonin (MT; 0, 0.05, 0.1 and 0.2 mM) on leaf relative water content; RWC (**A**), free amino acids; FAA (**B**), proline (**C**), reducing sugars (**D**), non-reducing sugars (**E**) and total soluble sugars (**F**) of potato plants grown under drought stress during the early stage of tuberization. Bars represent standard deviation (SD) of the means (*n* = 3). Different letters indicate significant differences among the treatments at *p* ≤ 0.05, according to Duncan’s multiple range test.

Exogenous MT exhibited a significant (*p* ≤ 0.05) improvement in leaf relative water content (RWC) free amino acids (FAA) compared to the untreated plants under both water stressed and non-stressed conditions (Figure 2A,B). In this respect, the highest significant results were obtained by the treatment of 0.1 mM MT under both irrigation conditions. Despite proline was not affected by different MT-treatments under well-watered conditions, an obvious and significant (*p* ≤ 0.05) decrease in proline was observed with increasing the concentration of MT under drought stress conditions (Figure 2C). Moreover, reducing, non-reducing and total soluble sugars were differentially affected by MT- treatments under both watering levels (Figure 2D–F). Under well-watered conditions, MT-treated plants displayed an obvious increase in reducing sugars. However, an opposite trend was observed in the non-reducing sugars. These changes slightly affected the concentration of total soluble sugars. In contrast, under drought stress, the general trend was that MT-treated plants at 0.05 and 0.1 mM exhibited an obvious increase in non-reducing sugars and total soluble sugars in parallel with a slight decrease in the reducing sugars. These findings imply that under drought stress condition, exogenous MT at the suitable concentrations (0.05 and 0.1 mM) may induce the sucrose biosynthesis (non-reducing sugar) which is considered the most transported form of soluble sugars. This effect may help potato plants to produce more tubers under water stress condition.

### 2.3. Effect of Melatonin on Cell Membrane Stability Index (CMSI), Methylglycoxal (MG), H_2_O_2_ and Lipid Peroxidation

**Figure 3:**
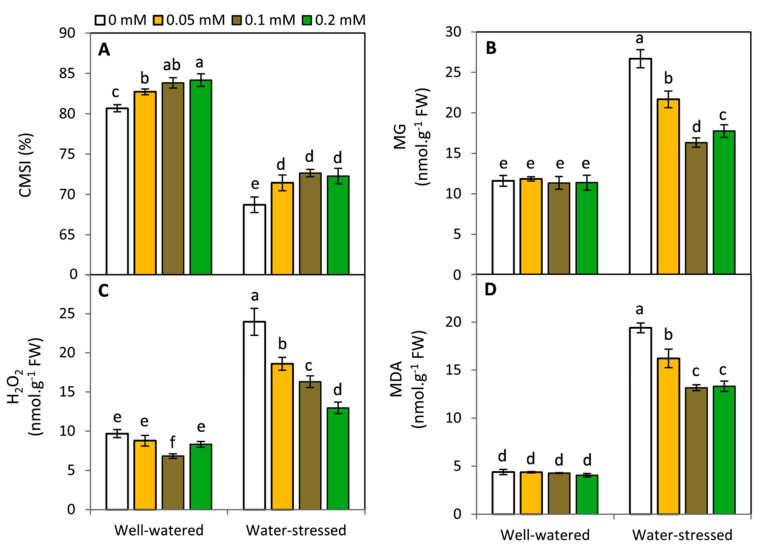
Effect of exogenous melatonin (MT; 0, 0.05, 0.1 and 0.2 mM) on cell membrane stability index; CMSI (**A**), methylglycoxal; MG (**B**), H_2_O_2_; (**C**), malondialdehyde; MDA (**D**) of potato plants grown under drought stress during the early stage of tuberization. Bars represent standard deviation (SD) of the means (*n* = 3). Different letters indicate significant differences among the treatments at *p* ≤ 0.05, according to Duncan’s multiple range test.

To evaluate the drought induced damages that occurred to plants in this study, CMSI, MG, H_2_O_2_, and MDA were estimated (Figure 3). Under well-watered condition, no significant changes were observed between all MT treatments in respect to MG and MDA. Meanwhile, MT at 0.2 and 0.1 mM resulted in the highest and lowest values of CMSI and H_2_O_2_; respectively. On the other hand, plants exposed to drought stress demonstrated a significant (*p* ≤ 0.05) decrease in CMSI, while, MG, H_2_O_2_ and MDA were significantly and dramatically increased in leaf tissues. Exogenous MT at all investigated concentrations significantly enhanced CMSI compared to the untreated plants either under well-watered or water-stressed conditions. On the contrary, MT-treated plants exhibited a significant decrease in MG, H_2_O_2_ and MDA under water deficit. In this respect, the lowest values were achieved by the treatments of MT at 0.1 and 0.2 mM compared to the untreated plants.

### 2.4. Effect of Melatonin on the Activities of Antioxidant Enzymes

**Figure 4:**
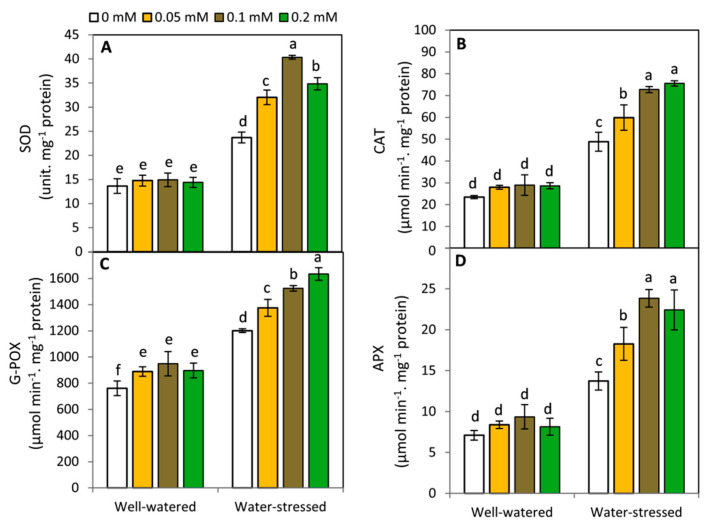
Effect of exogenous melatonin (MT; 0, 0.05, 0.1 and 0.2 mM) on superoxide dismutase; SOD (**A**), catalase; CAT (**B**), guaiacol peroxidase; G-POX (**C**), ascorbate peroxidase; APX (**D**) of potato plants grown under drought stress during the early stage of tuberization. Bars represent standard deviation (SD) of the means (*n* = 3). Different letters indicate significant differences among the treatments at *p* ≤ 0.05, according to Duncan’s multiple range test.

Regarding the antioxidant enzymes activities (Figure 4), it was observed that under water-stressed conditions, the activities of SOD, CAT, G-POX and APX were significantly (*p* ≤ 0.05) increased in potato plants compared to those of well-watered conditions. Moreover, the results showed that no significant differences were detected between MT-treated and non-treated plants in respect to SOD, CAT, and APX under well-watered conditions. Meanwhile, G-POX activity was increased in all MT-treated plants compared to the control under well-watered conditions. Under water shortage, applied MT led to a significant increase in the activities of all studied antioxidant enzymes. The highest activities of CAT and G-POX were obtained by the treatment of 0.2 mM MT, while, the treatment of 0.1 mM MT achieved the maximum findings in respect to SOD and APX.

### 2.5. Effect of Melatonin on ABA Concentration and Its Relationship with RWC

**Figure 5:**
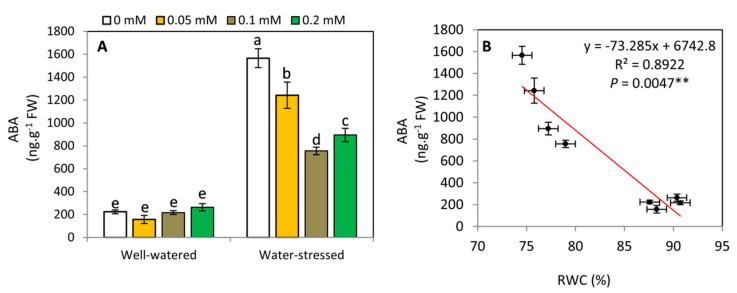
Effect of exogenous melatonin (MT; 0, 0.05, 0.1 and 0.2 mM) on abscisic acid; ABA (**A**), the relationship between ABA concentration and leaf relative water content; RWC (**B**) of potato plants grown under drought stress during the early stage of tuberization. Bars represent standard deviation (SD) of the means (*n* = 3). Different letters indicate significant differences among the treatments at *p* ≤ 0.05, according to Duncan’s multiple range test. ** (*p* ≤ 0.01).

The results of this study cleared that ABA was lower in potato plants under well-watered conditions compared with water-stressed conditions (Figure 5A). In addition, exogenous MT has no significant (*p* ≤ 0.05) effect on ABA content in potato under well-watered conditions. Under water-stressed conditions, ABA content was significantly affected by MT treatments. Also, 0.1 mM MT gave the lowest ABA content, followed by 0.2 mM, while the highest ABA content was recorded with control. Also, our results revealed that there is a linear and significant (*p* ≤ 0.01) negative correlation between RWC and ABA content in potato plants (Figure 5B).

### 2.6. Effect of Melatonin on Tuber Yield

**Figure 6:**
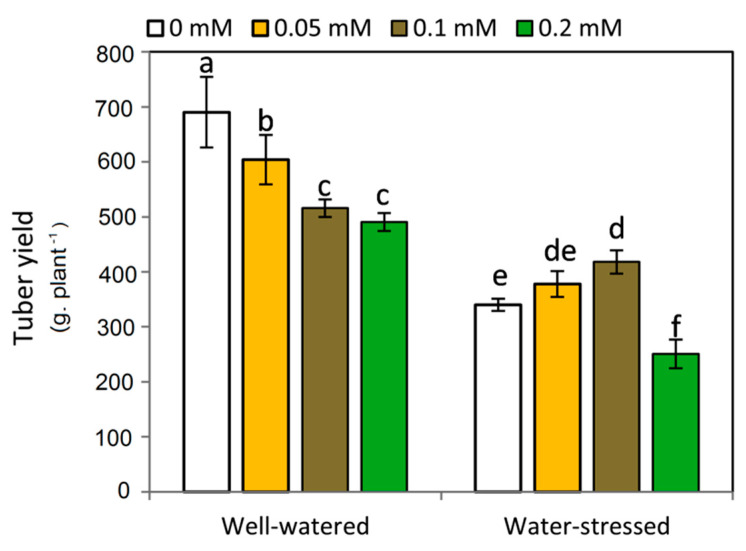
Effect of exogenous melatonin (MT; 0, 0.05, 0.1 and 0.2 mM) on tuber yield of potato plants grown under drought stress during the early stage of tuberization. Bars represent standard deviation (SD) of the means (*n* = 3). Different letters indicate significant differences among the treatments at *p* ≤ 0.05, according to Duncan’s multiple range test.

There was a highly significance difference between the effect of MT treatments on potato tuber yield under well-watered and drought stress conditions (Figure 6). Interestingly, applied-MT reduced the tuber yield compared to untreated plants under well-watered conditions, conversely, MT treatments up to 0.1 mM maximized tuber yield under water-stressed conditions. However, the treatment of 0.2 mM significantly inhibited tuber yield under drought stress condition.

## 3. Discussion

Drought is one of the devasting abiotic stresses that facing the cultivation of plants in throughout the world, limiting the growth and reducing the yield of plants. Drought sensitive vegetables, such as potato can be seriously affected by water shortage. Researchers around the world have made great efforts to overcome and alleviate the effect of this problem. In this context, some chemicals such as salicylic acid, jasmonic acid and γ-aminobutyric acid etc. have been extensively studied to induce drought tolerance in plants [47,48,49]. Similarly, melatonin as a new plant growth regulator has been suggested to mitigate a wide array of abiotic stresses [50]. Also, previous studies have revealed that exogenous melatonin can positively affect the productivity of various plant species [51,52,53]. In this study, the role of melatonin in alleviating water stress in potato plants was investigated. Melatonin treatment improved the fresh weight of potato plants and dry weight under water-stressed conditions compared with the untreated plants. These effects imply that exogenous melatonin as foliar application induced the tolerance to water stress in potato plants. Consistent with our results, Ye, et al. [54] found that melatonin improved the leaf area and shoot dry weight of maize seedlings under water stress. Moreover, the present study demonstrated that melatonin enhanced the chlorophyll content in potato plants under drought stress. Similarly, the activity of photosynthesis and photosystem II in rice seedlings were improved by exogenous melatonin. These responses were associated with increasing the activities of antioxidant enzymes leading to reduce the accumulation of ROS and MDA in plant cells [55]. Chlorophyll plays an important role in the light energy transmission and absorption [56], thereby synthesizing carbohydrates can be related to enhancing plant growth and productivity [57]. The results of this study indicated that 0.1 mM of melatonin was more effective in increasing chlorophyll and maximizing the yield of potato under water-stressed conditions. This effect could be attributed to increase the photosynthetic efficiency and protecting potato plants from drought induced oxidative damage. The physiological processes in the leaves of plant, such as transpiration, photosynthesis and respiration are regulated by leaf stomata through opening and closing which is affected by water balance and the pathways of complex signal transduction [8,45]. Relative water content (RWC) is an important physiological marker for the water status and plant’s ability to survive under drought stress conditions. In this study, water stress significantly reduced the RWC in the untreated plants. However, the exogenous melatonin as foliar application attenuated the reduction in RWC of treated potato. This suggests that melatonin improves the function of stomatal by stimulation of plant to reopen its stomata [28], and enhances the photosynthetic rate, allowing the RWC to increase under drought stress conditions. Also, Ahmad, et al. [51] found that length, width and area of stomata and pore numbers were reduced in maize seedlings under drought stress conditions compared to the control and MT-treated plants. In addition, the closure of stomata to prevent loss of water from the leaf by evapotranspiration may be due to the abscisic acid accumulation [58].

Several plant species, i.e., maize [59], tomato [27], green beans [1], and potato [7] can create a potential strategy to adapt stress conditions by maintaining osmotic potential. This effect can be occurred due to the accumulation of osmolyte substances, i.e., soluble sugars, free amino acids, soluble protein and proline. In this respect, in maize seedlings, proline has been found to scavenge OH^•^ radicals and quenches singlet oxygen to protect the membrane cells, protein and DNA from damage by ROS under abiotic stress [51]. The results of the present study showed that soluble sugars, free amino acids and proline content of MT-treated plants were significantly enhanced under water-stressed conditions compared to the untreated plants. These findings indicate that melatonin can stimulate the production of osmotic solvents to minimize water loss and protect the cell membrane under water-stressed conditions.

Exposure of plants to environmental stresses leads to generate the reactive oxygen species (ROS), including singlet oxygen (O^1^_2_), hydrogen peroxide (H_2_O_2_), hydroxyl radicals (OH), alkoxy radicals (RO), superoxide anion radicals (O^−^_2_). These toxic molecules may react with proteins, deoxyribonucleic acid and lipids triggering oxidative damage in plant cell [60]. In the same line, the results showed that hydrogen peroxide (H_2_O_2_) increased in potato under water-stressed conditions compared to the well-watered conditions. Meanwhile, exogenous melatonin significantly reduced H_2_O_2_ accumulation compared to the control under drought stress conditions. Also, a high significant reduction was detected in malondialdehyde (MDA) content in MT-treated plants compared to the untreated ones. Moreover, cell membrane stability index (CMSI) of potato was improved by MT treatments under water-stressed. Furthermore, the study of Li et al. [58] indicated that MT was effective in decreasing ROS under drought stress conditions. Therefore, the present study suggests that MT acts as an antioxidant and plays a vital role in overcoming the oxidative damage.

Methylglycoxal (MG) is an emerging signaling molecule in abiotic stress responses and tolerance in plants, is produced as a result of glycolysis in cells. Under normal conditions, MG remains at very low level. While, under stress conditions, MG accumulates to higher level. However, MG at low level, regulates the opening and closing stomata, reactive oxygen species production, concentration of cytosolic calcium ion and many stress-responsive genes expression. Whereas, MG high levels act as a toxic molecule, inhibits growth development processes, such as seed germination, root growth and photosynthesis, so, MG is considered as biochemical marker for abiotic stress tolerance in plants [61]. Our results indicated that MG significantly increased under water-stressed conditions, however, MT-treatment at a rate of 0.1 mM maintains MG at low level. This result cleared that 0.1 mM of melatonin is the best concentration to induce drought stress tolerance in potato plants.

Sueroxide dismutase (SOD), catalase (CAT), guiacol peroxidase (G-POX) and ascorbate peroxidase (APX) are essential protective enzymes related to the enzymatic defense system, effectively decomposing ROS and reducing H_2_O_2_ levels in plants [27,62,63]. Under stress conditions such as water deficit, plants evolve the mechanisms of tolerance to resist abiotic stress via activation several antioxidant enzymes, including SOD, CAT, G-POX and APX to protect the cell against oxidative damage [56]. The key of action mode of these enzymes is the balance between them, where SOD scavenges ROS and converts superoxide anion radicals (O^−^_2_) to O_2_ and H_2_O_2_, and then G-POX and CAT breakdown H_2_O_2_ to water [64]. In the present study, under water-stressed conditions, all antioxidant enzymes activities were significantly increased compared to the well-watered conditions. However, exogenous MT maximized the activities of SOD, CAT, G-POX and APX compared to control under water-stressed conditions. Whereas; H_2_O_2_ content decreased, the reduction of H_2_O_2_ may relate to antioxidant enzymes activity in MT-treated plants. Similarly, MT treatment improved CAT, SOD and G-POX activity in maize under drought stress [65]. These findings suggest that MT may play a crucial role in the activity of antioxidant enzymes and protecting plant cells from damage under drought stress.

Concerning the yield of potato, inevitably, water-stressed conditions had a negative effect and reduced the tuber yield of potato, but exogenous melatonin foliar application at a rate of 0.1 mM significantly increased tuber yield compared to control and another two concentrations. This indicates that melatonin has a vital role in tuber development under water stress by regulating the formation of growth regulators and non-reducing sugars. From the previous studies, melatonin and ABA function antagonistically or synergistically in order to regulate many processes in plants under stress conditions [58,66]. In this study, under drought stress, MT-treated plants showed a significant decrease in ABA compared to the untreated plants. This effect could be attributed to enhance leaf water status and antioxidant capacity of MT-treated plants. Several lines of evidence confirmed that, tuberization in potato plants is a developmental stage correlated with reducing of gibberellin (GA_3_) and increasing of sucrose. Also, there is an antagonism between ABA and GA_3_ in the presence of sucrose [43]. Indeed, ABA stimulates the tuber formation in potato via inhibition of stolon elongation, as prerequisite for formation process [67]. Also, there is a negative correlation between tuberization and reducing sugars content, while sucrose induces the tuberization by causing a hormonal changes in potato plants [68], sucrose induces the tuber-specific genes expression [69], acts as secondary messengers which have the ability to regulate the growth and development under abiotic and biotic stresses. Moreover, it is well documented that the photosynthetic carbon metabolites can be transported in plant as sucrose [70]. Hence, our study suggests that melatonin induces the potato tuberization and tubers formation and enhances tubers development under water-stressed conditions by increasing the non-reducing sugars and inhibition ABA transport from the root to shoot system leading to improving the tuber yield.

## 4. Material and Methods

### 4.1. Plant Material and Growth Conditions

Imported basic seed potatoes (*Solanum tuberosum* L.; CV, Hermes, Scotland) were kept for 3 weeks in a ventilated room until sprouting. After that, the homogenous seed tubers in size and form (35–40 mm) were planted in plastic pots (40 cm diameter) filled with 16 kg pre-washed sand (one sprouted tuber/pot). All pots were kept under greenhouse conditions during the period from 19 January to 10 May 2021. Air minimum and maximum temperatures and relative humidity (Table 1) were recorded inside the greenhouse using a digital Thermo/Hygrometer Art placed in the middle of the greenhouse (No.30.5000/30.5002, TFA; Wertheim, Baden-Württemberg, Germany).

### 4.2. Treatments and Experimental Layout

**Figure 7:**
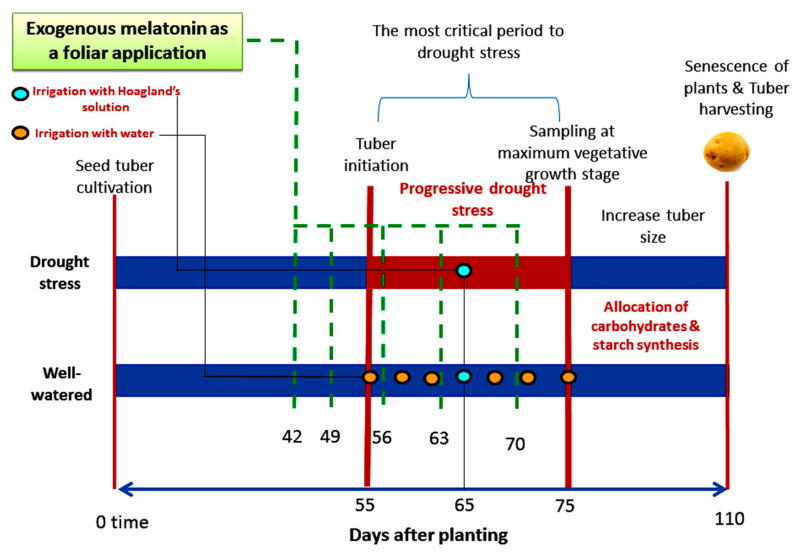
The timeline infographic for the treatments of melatonin as a foliar application and sampling of potato plants subjected to the well watered conditions and progressive drought stress during the tuberization stage.

To investigate the effect of exogenous melatonin (MT) as a foliar application on potato plants grown under well-watered and drought stressed conditions, plants were sparyed with MT (Bio Basic, Markham, York, Ontario, Canada) at 0.05, 0.1 and 0.2 mM. Addationally, distilled water was sprayed as a control. Tween-20 at 0.05% (*v/v*) as a wetting agent was used with all foliar treatments. The total number of pots (144) was divided into two major groups to apply the irrigation treatments (72 pots to apply the drought stress and 72 pots to apply the well-watered level). The timeline infographic for the treatments and sampling was shown in Figure 7. Each major group was divided into four subgroups to apply the different foliar treatments of MT (18 pots/subgroup). Each pot containing a single plant was weekly sprayed with 20 mL of specific MT concentration for 5 times at 42, 49, 56, 63, and 70 days after planting respectively. The progressive drought stress was applied during the most critical period of potato tuberization. Irrigation was stopped for 10 days (55–64 days after planting), rehydration was applied once with full strength Hogland’s solution at 65 days after planting; then irrigation was stopped again for 10 days (66–75 days after planting). However, the well-watered plants were irrigated seven times during this period (six times with tap water and one time with full strength Hogland’s solution as shown in Figure 7. The experimental layout was a Complete Randomized Design (CRD) including 3 replicates × 6 pots × 4 melatonin × 2 irrigation. Two pots from each replicate were left to the end of experiment at 110 days after planting to evaluate the tuber yield/plant.

### 4.3. Determination of Growth Parameters

When plants reached the maximum vegetative growth stage at 75 days after planting, two pots from each replicate were gathered to evaluate the growth parameters. Shoot fresh weight was immediately estimated after sampling using a digital balance, whereas shoot dry weight was determined by drying the samples in an air-forced ventilated oven at 105 °C.

### 4.4. Determination of Leaf Photosynthetic Pigments

The concentration of chlorophyll (Chl) a and b, and carotenoids in fresh leaves was determined spectrophotometrically according to Lichtenthaler and Wellburn [71]. Fresh weight (0.05 g) of fully expanded young leaves was used for pigment extraction in 80% acetone. The extract of pigments was measured versus a blank of pure 80% acetone at 663, 644, and 452.5 nm for Chla, Chlb, and carotenoid contents, respectively.

### 4.5. Determination of Relative Water Content (RWC), Osmolytes and Soluble Sugars

Leaf relative water content (RWC) was estimated according to Abd El-Gawad et al. [1]. Leaf discs from 6 of fully expanded leaves were weighed (FW) and placed immediately in distilled water for 2 h at 25 °C then the turgid weight (TW) was recorded. After that discs were fully dried in an oven at 110 °C for 24 h (DW). Relative water content (RWC) was calculated using the following formula:

$$\mathrm{RWC}\;\left(\%\right)=\frac{\mathrm{FW}-\mathrm{DW}}{\mathrm{TW}-\mathrm{DW}\;}\times 100$$

Free amino acids were determined using the ninhydrin reagent according to the method of Hamilton, et al. [72]. Proline concentration was determined by the method of ninhydrin reagent as described by Bates, et al. [73]. Total soluble sugars were estimated using the anthrone method [74], whereas, the reducing sugars were determined using the dinitrosalicylic acid method [75], and the non-reducing sugars content was estimated using the difference between the total soluble sugar content and the reducing sugars.

### 4.6. Determination of Cell Membrane Stability Index, Methylglyoxal, H_2_O_2_ and Lipid Peroxidation

Cell membrane stability was estimated as described by Abd Elbar, et al. [76] with some modifications. Eight leaf discs with 1.8 cm diameter were incubated in 10 mL deionized water for 24 h on a shaker. After that EC_1_ values of contents were measured by EC meters (DOH-SD1, TC-OMEGA, USA/Canada). Then, samples were autoclaved at 120 °C for 20 min to determine the values of EC_2_. Cell membrane stability index was calculated using the following equation:

$$\mathrm{MSI}=\left[1-\left(\frac{\mathrm{EC}1}{\mathrm{EC}2}\right)\right]\times 100$$

Methylglyoxal (MG) content was determined according to Hossain et al. [35] with some modifications. Fresh leaves (0.5 g) were homogenized in 3 mL of 0.5 M perchloric acid, then incubating for 15 min on ice. The mixture was centrifuged at 4 °C for 10 min at 10,000 rpm. The supernatant was decolorized by adding charcoal, kept for 15 min at room temperature, and centrifuged at 10,000 rpm for 10 min. Before using this supernatant for MG assay, it was neutralized by keeping for 15 min with saturated solution of potassium carbonate at room temperature and centrifuged again at 10,000 rpm for 10 min. The neutralized supernatant was used for MG estimation. One ml of the reaction mixture containing 250 μL of 7.2 mM 1, 2-diaminobenzene, 100 μL of 5 M perchloric acid, and 650 μL of the neutralized supernatant were added in that order. The absorbance at 335 nm was read after 25 min using a UV-spectrophotometer. Hydrogen peroxide (H_2_O_2_) concentration was estimated colorimetrically by the method of potassium iodide [77]. Malondialdehyde (MDA) was determined by thiobarbituric acid (TBA) method [78].

### 4.7. Assay of Antioxidant Enzymes and ABA Determination

Fresh leaves (0.2 g) were ground in 4 mL of 0.1 M ice-cold sodium phosphate buffer (pH 7.0) containing 1% (*w/v*) polyvinylpyrrolidon (PVP) and 0.1 mM EDTA, centrifuged at 10,000× *g* at 4 °C for 20 min. The supernatant was used for the next enzyme activity assays. To calculate the specific activity of different enzymes, total soluble protein was also determined in the supernatant according to Bradford [79]. Superoxide dismutase (SOD; EC 1.15.1.1) activity was evaluated according to the ability to inhibit the photochemical reduction of nitro blue tetrazolium (NBT) at 560 nm [80]. Catalase (CAT; EC 1.11.1.6) activity was assayed by monitoring the decrease in absorbance of H_2_O_2_ at 240 nm [81]. Guaiacol peroxidase (G-POX; EC1.11.1.7) activity was evaluated by observing its ability to convert guaiacol to tetraguaiacol by monitoring the increase in absorbance at 470 nm [82]. Ascorbate peroxidase (APX; EC 1.11.1.11) activity was determined based on the decrease in ascorbate at 290 nm [83]. Abscisic acid (ABA) was extracted from fresh leaf tissues according to the method of Shindy and Smith [84]. Then, High-Performance Liquid Chromatography (HPLC; SCIEX, Framingham, MA, USA) was used to complete the quantification procedure according to Nakurte, et al. [85].

### 4.8. Statistics

One way ANOVA procedure was followed using SAS [86] software. Means ± SD were calculated from three replicates and Duncan’s multiple range test (*p* ≤ 0.05) was used to determine the significant differences between means.

## 5. Conclusions

**Figure 8:**
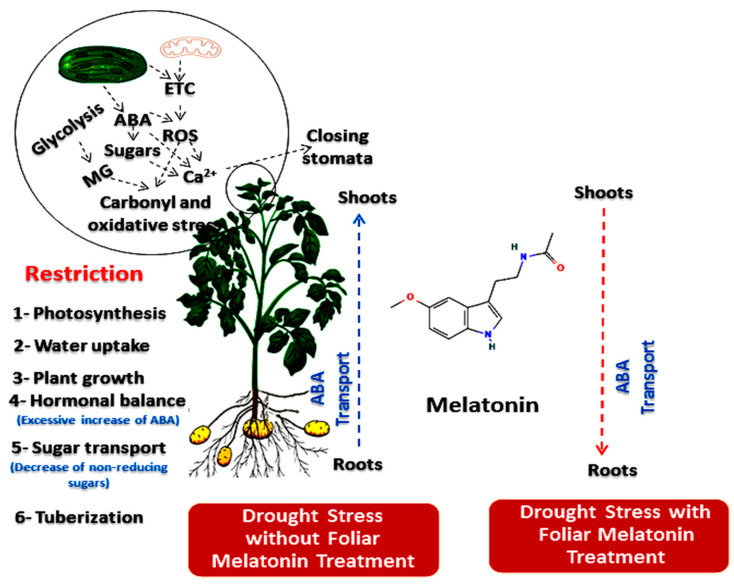
Simplified model for the suggested effect of exogenous melatonin as foliar spray on potato plants grown under drought stress during the early stage of tuberization. In drying soil, ABA that is synthesized in roots can be carried by xylem stream to the shoot system leading to stomata closing and reducing the rate of transpiration. Moreover, soluble sugars tends to accumulate in shoots as reducing sugars (not suitable form to sugar transport) leading to decrease the rate of tuberization. Conversely, melatonin treatments led to decrease ABA in shoots as resulting to enhancement of leaf water status and antioxidant capacity. This effect may also be explained by restriction of ABA transport from root to the shoot system. Furthermore, MT-treated plants tended to accumulate the non-reducing sugars (the most suitable form to sugar transport and starch synthesis) leading to improve the tuberization. ETC, electron transport chain in chloroplast and mitochondria; ABA, abscisic acid; MG, methylglyoxal; ROS, reactive oxygen species.

In conclusion, this study demonstrated the beneficial effects of melatonin (MT) in alleviating drought stress during the most critical period of early tuberization. Applied-MT decreased the harmful effects of drought induced carbonyl/oxidative stress. These effects were observed by reducing the cytotoxic biochemical cellular markers (MG, H_2_O_2,_ and MDA). These responses were in parallel with enhancing plant growth, chlorophyll content, leaf water status and activities of antioxidant enzymes. Moreover, MT can differentially regulate the tuberization process under well-watered and drought stressed conditions. This regulation may be correlated with the effect of MT on increasing the non-reducing sugars on one side; and restriction of ABA transport from the root to shoot system on the other side (Figure 8). Further studies using advanced molecular techniques are required to better understand the role of MT in tuberization process and inducing different signaling pathways in potato plants under water deficiency.

## Figures and Tables

**Table 1:** Summary of the monthly mean climate condition, maximum (T_max_) and minimum (T_min_), mean (T_ave_) temperatures and relative humidity (RH), inside the greenhouse.

Month	T_max_	T_min_	T_ave_	RH (%)
January	23.9	11.4	17.6	62.6
Febrauary	26.2	13.1	19.6	65.2
March	27.4	13.9	20.6	65.7
April	33.7	15.6	24.6	60.2
May	38.5	24.2	31.4	58.6

## Data Availability

Not applicable.
